# Sphingosine Kinase-1 Involves the Inhibitory Action of HIF-1α by Chlorogenic Acid in Hypoxic DU145 Cells

**DOI:** 10.3390/ijms18020325

**Published:** 2017-02-04

**Authors:** Myoung-Sun Lee, Seon-Ok Lee, Kyu-Ri Kim, Hyo-Jeong Lee

**Affiliations:** 1 College of Korean Medicine, Kyung Hee University, 1 Hoegi-dong, Dongdaemun-gu, Seoul 130-701, Korea; lmsms14@naver.com (M.-S.L.); lso4595@naver.com (S.-O.L.); 2 Graduate School of East-West Medical Science, Kyung Hee University; 1 Hoegi-dong, Dongdaemun-gu, Seoul 130-701, Korea; oknice79@gmail.com

**Keywords:** chlorogenic acid, sphingosine kinase-1, hypoxia, prostate cancer

## Abstract

Hypoxia enhances cancer development in a solid tumor. Hypoxia-inducible factor-1 α (HIF-1α) is a transcription factor that is dominantly expressed under hypoxia in solid tumor cells and is a key factor that regulates tumor. HIF-1α regulates several target genes involved in many aspects of cancer progression, including angiogenesis, metastasis, anti-apoptosis and cell proliferation as well as imparts resistance to cancer treatment. In this study, we assessed *Crataegus Pinnatifida* Bunge var. typical Schneider ethanol extract (CPE) for its anti-cancer effects in hypoxia-induced DU145 human prostate cancer cell line. CPE decreased the abundance of HIF-1α and sphingosine kinase-1 (SPHK-1) in hypoxia-induced prostate cancer DU145 cells. CPE decreased HIF-1α and SPHK-1 as well as SPHK-1 activity. Chlorogenic acid (CA) is one of four major compounds of CPE. Compared to CPE, CA significantly decreased the expression of HIF-1α and SPHK-1 as well as SPHK-1 activity in hypoxia-induced DU145 cells. Furthermore, CA decreased phosphorylation AKT and GSK-3β, which are associated with HIF-1α stabilization and affected SPHK-1 in a concentration-dependent manner. We confirmed the mechanism of CA-induced inhibition of HIF-1α by SPHK-1 signaling pathway using SPHK-1 siRNA and SPHK inhibitor (SKI). CA decreased the secretion and cellular expression of VEGF, thus inhibiting hypoxia-induced angiogenesis. Treatment of DU145cells with SPHK1 siRNA and CA for 48 h decreased cancer cell growth, and the inhibitory action of SPHK siRNA and CA on cell growth was confirmed by decrease in the abundance of Proliferating cell nuclear antigen (PCNA).

## 1. Introduction

The fruit of *Crataegus pinnatifida* (Shanzha) [1] is used in traditional Oriental medicine. Shanzha is widely distributed in Asia and Europe [2,3] and is commonly used to treat cardiovascular diseases [4], cataract [5], asthma [6], and indigestion [1]. Previous reports demonstrated that Shanzha extract exhibits anti-tumorigenic [7], anti-fatty liver [3], anti-diabetic [8], anti-atherosclerosis [9], and antioxidative [10] effects. However, the effect of the Shanzha under hypoxia remains unclear. A previous study reported an important chemical component of *Crataegus pinnatifida* [11]. This component of *Crataegus pinnatifida* obtained from ethanol extraction includes four major compounds: Chlorogenic acid (CA), hyperoside, iso-quercetin and procyanidin-B2 [10,12]. In this study, we discuss the effects of CA in hypoxia-induced prostate cancer.

One of the characteristics of solid tumor is hypoxia [13]. It is a condition in which the tissues are not oxygenated adequately and is associated with resistance to radiotherapy and chemotherapy. Hypoxia-inducible factor-1α (HIF-1α) is a transcription factor that regulates various biological processes under hypoxia in cancer, such as metabolism, cell proliferation and migration, angiogenesis and apoptosis. Thus, HIF-1α is an important target for cancer therapy [14]. A recent study showed that the activation Ras induces the expression of HIF-1α involved [15,16]. HIF-1α, when stabilized by hypoxic conditions, mediates the response to hypoxia and upregulates many genes important for cancer development such as a vascular endothelial growth factor (VEGF) which promotes angiogenesis [14]. Sphingosine and sphingosine 1-phosphate (S1P) regulates various biological processes, including cell proliferation, apoptosis, and angiogenesis. Sphingosine kinase-1(SPHK-1) catalyzes the phosphorylation of sphingosine to form S1P. SPHK-1 is known to regulate HIF-1α expression under hypoxia [17], and it is reported that SPHK-1 is a new target for cancer therapy [18]. SPHK-1 activates the AKT/GSK-3β signaling pathway, which is involved in the accumulation of HIF-1α levels under hypoxia in cancer [19]. Thus, in hypoxic tumors, HIF-1α regulates many genes involved in cancer development and SPHK-1 regulates and stabilizes HIF-1α through the AKT/GSK-3β pathway. However, under normoxia the polyubiquitylation of HIF-1α by Von Hippel-Lindau syndrome (VHL) degraded HIF-1α in proteasome [18].

CA is found in natural products such as coffee [20]. It regulates various biological processes and has anti-inflammatory [21], anti-diabetic [22], anti-tumorigenic [23], antioxidative [24], anti-gout [25], and anti-obesity [26] effects. Recently, it has been shown that CA inhibits HIF-1α mRNA expression [27] and angiogenesis through the AKT pathway [28]. However, the mechanisms underlying the CA-mediated inhibition of HIF-1α through the SPHK-1 pathway under hypoxia are still not well understood. Thus, in this study, we evaluated whether the inhibition of HIF-1α by CA involves the SPHK-1 pathway under hypoxia in the DU145 human prostate cancer cell line.

## 2. Results

### 2.1. CPE Decreases HIF-1α and SPHK-1 Abundance in Hypoxic Condition

**Figure 1:**
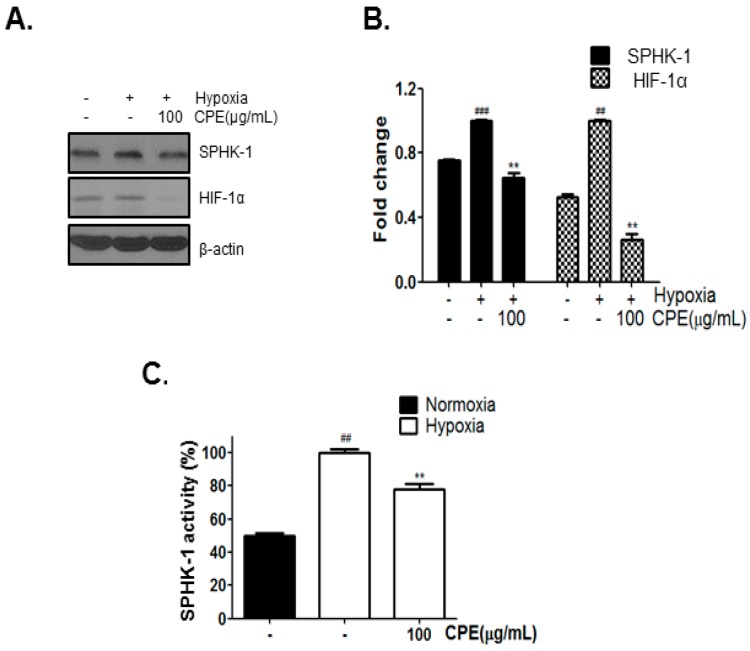

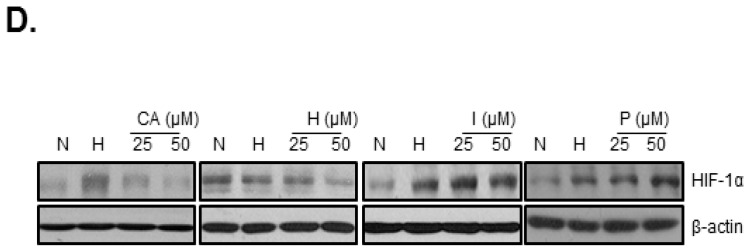
Inhibitory effect of CPE containing CA on HIF-1α expression in hypoxic DU145 cells. (**A**) DU145 cells were treated with CPE (0 and 100 μg/mL) for 4 h. Levels of SPHK-1, HIF-1α, and β-actin expression were determined by western blot analysis; (**B**) Fold change of western blot. Data are presented as means ± S.D. (**) *p* < 0.01 compared to control under hypoxia. (###) *p* < 0.001, (##) *p* < 0.01, compared to control under normoxia; (**C**) DU145 cells were treated with CPE (0, and 100), SPHK-1 activity was measured by using SPHK-1 activity assay kit. Data are presented as means ± S.D. (##) *p* < 0.01 compared to control under normoxia. (**) *p* < 0.01 compared to control under hypoxia; (**D**) Effect of four major compounds of *Crataegus pinnate* *fida* on hypoxia-induced HIF-1α expression in DU145 cells. (N: Normoxia, H: Hypoxia, CA: Chlorogenic acid, H: Hyperoside, I: Isoquercetin, P: Procyanidin B2).

According to our precedent data, *Crataegus Pinnatifida* Bunge var. typical Schneider ethanol extract (CPE) more than 10% decreased DU145 cell growth under hypoxic condition compared to under normoxic condition (data not shown). To investigate whether CPE affects the expression of HIF-1α and SPHK-1, DU145 cells were incubated with 100 µg/mL CPE for 4 h in hypoxic condition. As shown in Figure 1A–C, CPE decreased hypoxia-induced expression of SPHK-1 and HIF-1α as well as SPHK-1 activity. Thus, the abundance of HIF-1α and SPHK-1 increased in hypoxic condition compared to in normoxic condition.

### 2.2. Chlorogenic Acid (CA), One of Four Major Compounds of CPE, Decreases HIF-1α and SPHK-1

**Figure 2:**
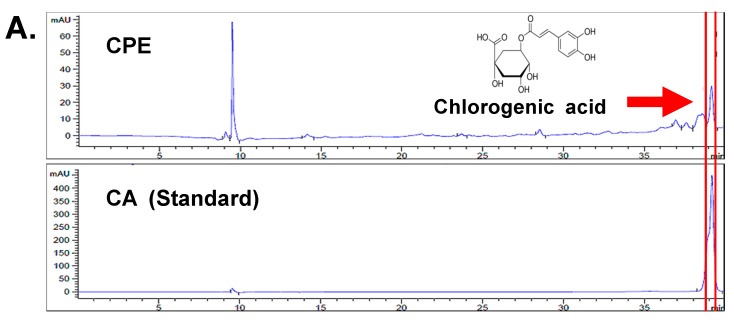

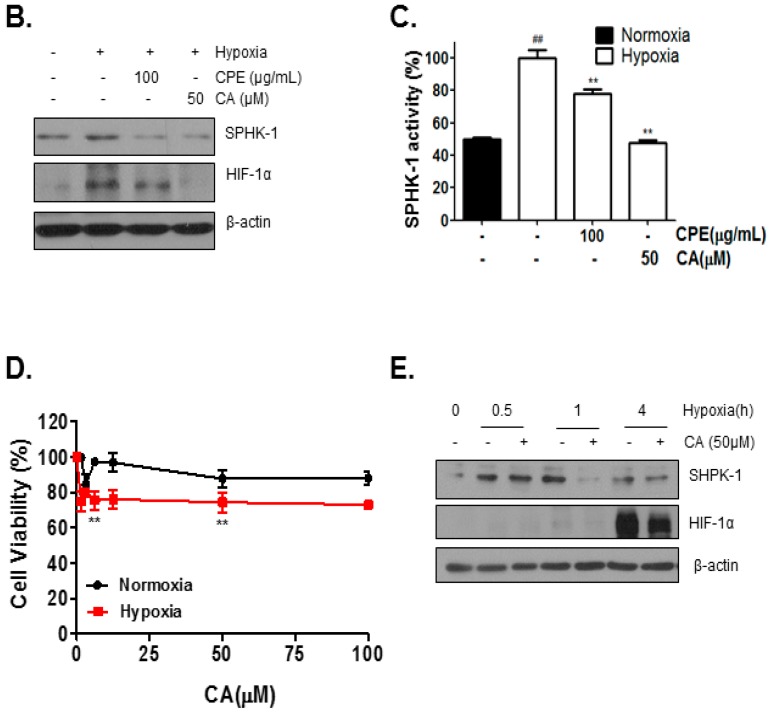
Effect of CA on hypoxia-induced SPHK-1 and HIF-1α expression and SPHK-1 activity in DU145 cells. (**A**) Upper graph: HPLC chromatograms of CPE. Lower graph: Standard peak of CA (39.172 min); (**B**) Effect of CA on hypoxia-induced SPHK-1 and HIF-1α expression; (**C**) Inhibitory effect of CA on hypoxia-induced SPHK-1 activity assay. Data are presented as means ± S.D. (**) *p* < 0.01 compared to control under hypoxia. (##) *p* < 0.01, compared to control under normoxia; (**D**) Effects of CA on the cytotoxicity of DU145 cells for 24 h under normoxic and hypoxic condition. Data are presented as means ± S.D. (**) *p* < 0.01 compared to control; (**E**) Change of SPHK-1 and HIF-1α expression by hypoxic exposure time.

To determine the inhibitory effect of the four major compounds (Chlorogenic acid (CA)**,** Hyperoside (H), Isoquercetin (I), Procyanidin B2 (P)) of *Crataegus Pinnatifida* Bunge var. *typica* Schneider on HIF-1α, DU145 cells were incubated with each of these four compounds in hypoxic condition for 4 h. The results showed that CA and hyperoside decreased the expression of HIF-1α (Figure 1D); however, the inhibitory effect of CA on the expression of HIF-1α was higher compared to the inhibitory effect of hyperoside. High-performance liquid chromatograpy (HPLC) was performed to confirm whether CPE contains CA. As shown in Figure 2A, CPE was found to contain CA and the peak shows that the retention time of CA was 39 min. Compared to CPE, CA significantly decreased the hypoxia-induced expression of HIF-1α and SPHK-1 as well as SPHK-1 activity (Figure 2B,C).

To measure whether CA affects cell viability under hypoxic and normoxic conditions, DU145 cells were treated with various concentrations of CA under hypoxia or normoxia for 24 h. We found that CA significantly decreased cell viability under hypoxia compared to normoxia (Figure 2D).

In the time-dependent study of the expression of SPHK-1 and HIF-1α after exposure to hypoxia, SPHK-1 expression increased at an early time (0.5 h) whereas HIF-1α expression peaked at 4 h. after hypoxia exposure. On the other hand, CA suppressed this increase in the expression of SPHK-1 and HIF-1α in hypoxia- induced DU145 cells (Figure 2E).

### 2.3. CA Inhibits Phosphorylation of AKT and GSK-3β, Which Are Involved in HIF-1α Stabilization, by SPHK-1

**Figure 3:**
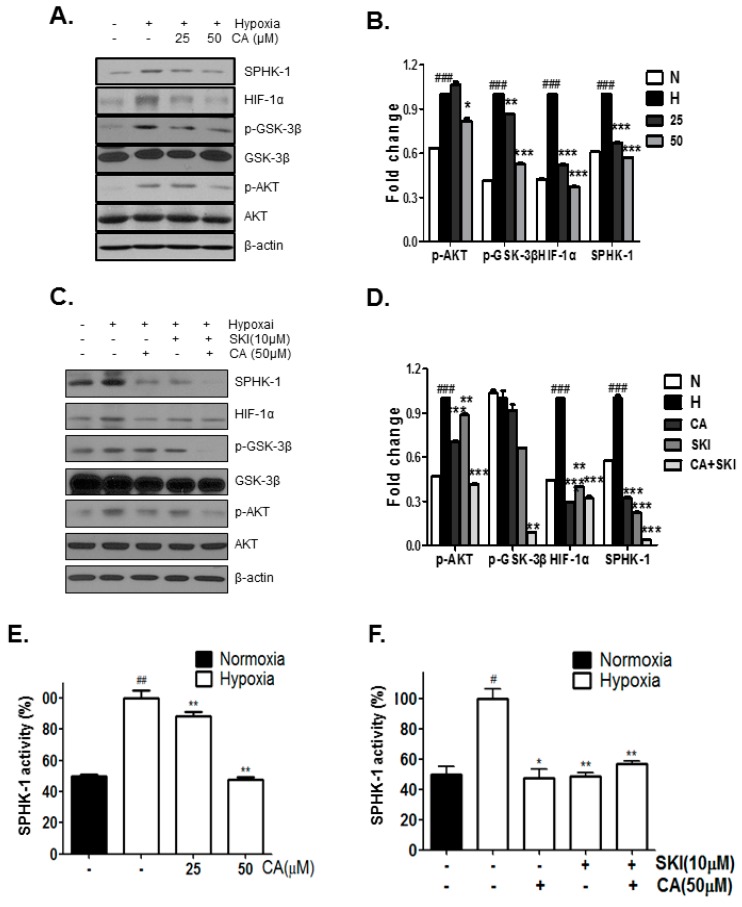
Effect of CA on hypoxia-induced SPHK-1 signaling in DU145 cells. DU145 cells were treated without or with CA (25 and 50 μM) under hypoxia for 4 h. Levels of phosphorylated and total AKT and GSK-3β, SPHK-1, and HIF-1α proteins were determined by western blot analysis. SPHK-1 activity was determined by SPHK-1 activity assay. (**A**) Levels of SPHK-1, HIF-1α, AKT, p-AKT, GSK-3β, p-GSK-3β and β-actin by CA; (**B**) Quantitative protein levels are shown. The results are expressed as means ± S.D. (###) *p* < 0.001, compared to control under normoxia. (*) *p* < 0.05, (**) *p* < 0.01, (***) *p* < 0.001, compared to control under hpoxia; (**C**) Inhibitory effect of SKI on hypoxia–induced SPHK-1 signaling in DU145 cells; (**D**) Quantitative protein levels are shown. The results are expressed as means ± S.D. (###) *p* < 0.001, compared to control under normoxia. (**) *p* < 0.01, compared to control under hypoxia; (**E**) SPHK-1 activity. Data are presented as means ± S.D. (**) *p* < 0.01 compared to control under hypoxia. (##) *p* < 0.01, compared to control under normoxia; (**F**) Effect of SKI on hypoxia-induced SPHK-1 activity. Data are presented as means ± S.D. (*) *p* < 0.05, (**) *p* < 0.01 compared to control under hypoxia. (#) *p* < 0.05, compared to control under normoxia.

To investigate whether the decrease in HIF-1α expression by CA in hypoxic prostate cancer cells involves in the SPHK-1 pathway, AKT, and GSK-3β and to delineate the downstream signaling mechanism of SPHK-1, we performed western blot analysis and SPHK-1 activity assay. SPHK-1, HIF-1α, p-AKT, and p-GSK-3β were increased in hypoxic control compared to the normoxic control, whereas CA inhibited the hypoxia-induced expression of SPHK-1, HIF-1α, p-AKT, and p-GSK-3β (Figure 3A). Consistent with western blotting results, CA inhibited the hypoxia-induced activity of SPHK-1 in the SPHK-1 activity assay. The rate of inhibition of SPHK-1 activity by CA at 25 μM and 50 μM were 12% and 50%, respectively (Figure 3B). Similar to this action of CA, sphingosine kinase inhibitor (SKI) inhibited the expression of SPHK-1, HIF-1α, pAKT, and pGSK-3β. Combination treatment with SKI and CA suppressed the expression and activity of SPHK-1 as well as the expression p-AKT, p-GSK-3β, and HIF-1α in hypoxia-induced DU145 cells (Figure 3C,D).

### 2.4. CA Inhibits Hypoxia-Induced Angiogenesis

**Figure 4:**
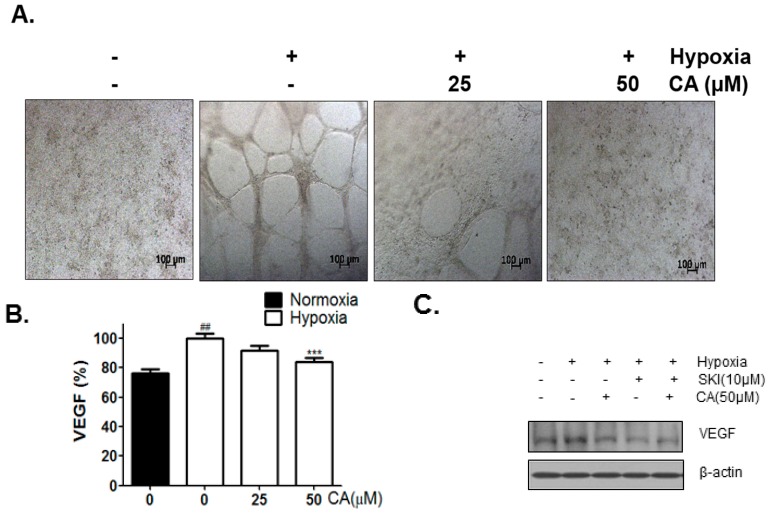
Inhibitory effect of CA on angiogenesis in DU145 cells under hypoxia for 24 h. (**A**) Tube formation assay using human umbilical vein endothelial cells (HUVECs). HUVECs on matrigel were treated conditioned medium for 24 h. Scale bars = 100 μm; (**B**) VEGF (%) was measured by using VEGF assay kit. Data are presented as means ± S.D. (##) *p* < 0.01 compared to control under normoxia. (***) *p* < 0.001 compared to control under hypoxia; (**C**) DU145 cells were treated without or with CA (25, and 50 were treated hours under hypoxia and subjected to western blot analysis of protein levels (VEGF, and β-actin).

To analyze the anti-angiogenic effect of CA, Human umbilical vein endothelial cells (HUVECs) tube formation assay was performed. The supernatants of the culture media from the hypoxia-induced DU145 cells treated with different concentrations of CA (0, 25, and 50 µM) for 24 h were used for HUVECs tube formation. As shown in Figure 4A, hypoxia-induced supernatant from cells without treatment with CA formed the HUVEC tube well, whereas hypoxia-induced supernatant form cells treated with CA suppressed the HUVECs tube formation. Vascular endothelial growth factor (VEGF) content of the supernatant was analyzed by VEGF enzyme-linked immunosorbent assay (ELISA). Consistent with the results of HUVECs tube formation assay, CA suppressed the secretion of VEGF. The rate of inhibition of VEGF activity by CA at 25 μM and 50 μM was 10% and 18%, respectively (Figure 4B). VEGF cellular level was checked by western blotting. Both SKI and CA were found to inhibit VEGF expression (Figure 4C).

### 2.5. CA Inhibits Cell Proliferation in Hypoxic Condition

**Figure 5:**
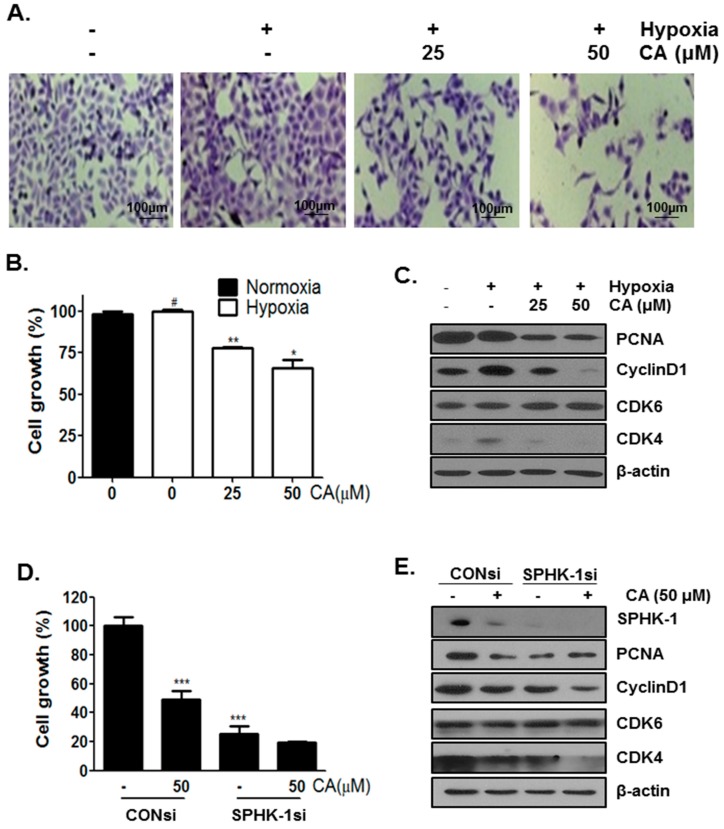
Inhibitory effect of CA on proliferation in DU145 cells under hypoxia for 3 days. (**A**) DU145 cells were treated without or with CA (25, and 50 µM) for 3 days under hypoxia and subjected to Crystal violet staining of cell growth. Scale bar = 100 μm ; (**B**) DU145 cells resolved in 70% ethanol after Crystal violet staining, and the optical density was read using a microplate reader at 570 nm. Data are presented as means ± S.D. (#) *p* < 0.05 compared to control under normoxia, (**) *p* < 0.01 and (*) *p* < 0.05 compared to control under hypoxia; (**C**) DU145 cells were treated without or with CA (25, and 50 µM) for 3 days under hypoxia and subjected to western blot analysis of protein levels (PCNA, CyclinD1, CDK6, CDK4, and β-actin); (**D**) DU145 cells were treated without or with CA (50 μM) for 3 days under hypoxia after SPHK-1 silencing for 24 h and subjected to Crystal violet staining of cell growth. DU145 cells resolved in 70% ethanol after washing with distilled water, and the OD was read using a microplate reader at 570 nm. Data are presented as means ± S.D. (***) *p* < 0.001 compared to control under hypoxia; (**E**) DU145 cells were treated without or with CA (50 µM) for 3 days under hypoxia after SPHK-1 silencing for 24 h and subjected to western blotting (SPHK-1, PCNA, CyclinD1, CDK6, CDK4, and β-actin).

To investigate whether the inhibition of HIF-1α expression affects cancer cell growth, DU145 cells were treated with different concentrations of CA (0, 25, and 50 μM) under hypoxia for 3 days. Cell morphology showed a decrease in cell number in the CA-treated group (Figure 5A,B). Treatment of hypoxia-induced DU145 cells with CA for 3 days caused a G1 cell cycle arrest(Supplementary Figure S1) and the G1- related proteins were checked by western blotting. CA treatment resulted in a decrease in PCNA, cyclinD, and cyclin-dependent kinase-4 (CDK4) levels but did not affect cylin-dependent kinase-6 (CDK6).

To investigate whether the anti-proliferative effect of CA involves SPHK-1, hypoxia-induced DU145 cells were treated with SPHK-1 siRNA and CA for 3 days. As shown in Figure 5D,E, a decrease in cell viability was observed in SPHK-1 siRNA-transfected cells compared to in the control siRNA-transfected cells. Consistent with the above result, in the CA- treated group, SPHK-1 siRNA inhibited the expression of PCNA, cyclin D1, and CDK4 but did not affect the expression of CDK6.

## 3. Discussion

We provide the first evidence of anti-cancer effects of CPE and the regulatory mechanism of anti-cancer effects of its component, CA.

CA is a polyphenol found in various natural products, such as coffee and apple. It has a wide range of biological properties, including anti-inflammatory, anti-oxidative, anti-neurotoxic, and anti-cancer properties [29,30,31,32]. A recent study reported that CA suppressed HIF-1α and hypoxia-induced angiogenesis in A549 lung cancer cells [28] and that the mechanisms of the inhibition of hypoxia-induced HIF-1α by CA involve stabilization of HIF-1α by the AKT pathway. However, they did not discuss the mechanisms upstream of AKT, which are involved in the stabilization of HIF-1α. There are two representative kinases, phosphatidylinositol-4,5-bisphosphate3-kinase (PI3K) and SPHK-1, upstream AKT. These kinases stablize HIF-1α by regulating of AKT in hypoxic cancer cells [33]. Particularly, PI3K/AKT pathway is better understood than SPHK-1 pathway in a hypoxic environment in cancer development. In this study, we report that CA and SKI did not affect PI3K levels in hypoxia-induced DU145cells (Supplementary Figure S1). Similar to CA, SKI did not affect PI3K levels too (Supplementary Figure S1); therefore, CA affects the accumulation of HIF-1α levels through inhibition of SPHK-1 pathway and not PI3K.

It is well known that solid tumors are subject to hypoxia, and it is important for the regulation of several signals in cancer cells which make them more aggressive and resistant to radiotherapy and chemotherapy [34]. SPHK and PI3K regulate HIF-1α by inhibiting von Hippel-Linadu tumor suppressor (pVHL)-dependent proteasomal degradation. PI3K and SPHK-1 pathway have been implicated in cancer chemoresistance [35]. Their downstream signaling involves AKT/mTOR(mechainistic target of rapamycin) pathway that leads to chemoresistance mediated by HIF-1α [36]. A recent study reported that SPHK-1 overexpression can block the effect of the mTOR inhibitor and induce the reactivation of the mTOR/HIF-1α pathway [37]. Therefore, these pathways regulating HIF-1α are a potential therapeutic target for cancer.

In the present study, the inhibition of SPHK-1 activity using SKI prevented VEGF cellular expression in hypoxia-induced DU145 cells (Figure 4C). This finding is consistent with previous studies that have demonstrated that SPHK-1 plays a critical role in HIF-1α-mediated VEGF levels under hypoxia [19,38,39].

CA decreased cell viability in hypoxic cancer cells (Figure 2D) as well as cell proliferation for 72 h (Figure 5). It is well known that SPHK-1 expression is related to cancer cell proliferation and progression. To confirm the role of SPHK-1 in CA-mediated inhibition of cell proliferation, hypoxic DU145 cells were treated with SKI siRNA and CA for 72 h. Interestingly, SKI and CA co-treatment decreased the expression of CDK4 and cyclin D1 in hypoxic DU145 cells.

## 4. Materials and Methods

### 4.1. Test Compound

Chlorogenic acid (CA) (purity ≥ 95% as determined by HPLC) was purchased from Sigma-Aldrich (St. Louis, MO, USA).

### 4.2. Cell Culture Assay

DU145 cells were purchased from Korean Cell Line Bank (KCLB) (Seoul, Korea) and maintained in RPMI1640 medium (Welgene, Daegu, Korea), supplemented with 10% fetal bovine serum (Welgene, Daegu, Korea) and penicillin/streptomycin (WelGene, Daegu, Korea) at 37 °C with 5% CO_2_ in a humidified incubator.

### 4.3. Hypoxia Treatment

Cells cultured under hypoxia were grown in an anaerobic chamber (FormaScientific, Marietta, OH, USA) flushed with a custom gas mixture containing 1% oxygen, 5% carbon dioxide and 94% nitrogen at 37 °C.

### 4.4. Cytotoxicity Assay

Cytotoxicity of CPE and CA were evaluated by 3-(4,5-dimethylthiazol-2-yl)-2,5-diphenyl tetrazolium bromide (MTT) (Sigma-Aldrich, St. Louis, MO, USA) assay. Cells were seeded at a density of 1 × 10^4^ cells per well in a 96 well plate, cultured for 24 h, and then treated with various concentrations of CPE and CA under hypoxia and normoxia. After 24 h incubation, the 50 µL of MTT solution (1 mg/mL) was added to each well and incubated at 37 °C in dark for 2 h. The viable cell number was correlated with the production of formazan that was dissolved in dimethyl sulfoxide (DMSO) and optical density (OD) was measured at 570 nm using a microplate reader (Molecular Devices Co., Sunnyvale, CA, USA). Cell viability was calculated by the following equation: Cell viability (%) = [OD (sample) − OD (blank)]/[OD (control) − OD (blank)] × 100.

### 4.5. Western Blot Analysis

DU145 cells (5 × 10^5^ cells) were cultured in the without or with of CA or CPE. Cells were lysed in RIPA buffer (50 mM Tris-HCL, pH 7.4, 150 mM NaCl, 1% NP-40, 0.25% deoxycholic acid-Na, 1 M EDTA, 1 mM Na_3_VO_4_, 1 mM NaF and protease inhibitors cocktail). Protein samples were quantified by using a Bio-Rad DC protein assay kit II (Bio-Rad, Hercules, CA, USA), separated by electrophoresis on 8% SDS-PAGE gel and electrotransferred onto a Bio Trace NT transfer membrane (pall, Gelman Laboratory, Port Washington, NY, USA). The membranes were blocked in 3% nonfat skim milk and probed with primary antibodies for SPHK-1 (Cell Signaling, Denvers, MA, USA), HIF-1α (BD, San Jose, CA, USA), AKT (Santa Cruz Biotechnology, Santa Cruz, CA, USA), p-AKT (Santa Cruz Biotechnology), GSK-3β (Cell Signaling), p-GSK-3β (Cell Signaling), β-actin (Sigma-Aldrich) overnight and exposed to horseradish peroxidase (HRP)-conjugated secondary anti-mouse or rabbit antibodies. Protein expression was examined by using EZ-Western Lumi Pico (DOGEN, Seoul, Korea).

### 4.6. HPLC Analysis

To analyze the components of CPE, CA were run on Hichrom HPLC columns (5 μm, 250 × 4.6 mm, Hichrom, Ltd., Theale, UK) using a high HPLC system (Agilent Technologies, Santa Clara, CA, USA). The mobile phase consisted of 95% water containing 0.1% trifluoroacetic acid (solvent A) and 5% methanol containing 40% acetonitrile (solvent B). The solvent flow rate was 0.3 mL/min with gradient elution: 0–31.2 min, 28.4%; 31.2–31.4 min, 30.4%; 31.4–38 min, 34.7%; 38–50 min, 45% of solvent B. The ambient temperature was set at 45 °C. UV detection wavelength was set at 230 nm.

### 4.7. Crystal Violet Staining

DU145 cells were seeded into 6-well plates and treated with or without of CA under hypoxia or normoxia. After 3days, cells were washed with PBS, fixed 1% glutaraldehyde in PBS for 20 min at room temperature and stained with 0.05% crystal violet solution. After washing with distilled water, cells were resolved in 70% ethanol and the O.D. was read at 590 nm using a microplate reader (Molecular Devices Co., Sunnyvale, CA, USA).

### 4.8. Sphingosine Kinase Activity Assay

Sphingosine kinase activity was measured by using sphingosine kinase activity assay kit (Echelon, Salt Lake City, UT, USA) according to the manufacturer’s instructions. In brief, protein extracts (30 μg) were incubated in reaction buffer (100 µM sphingosine and 10 µM ATP) for 1 h at 37 °C, and luminescence attached ATP detector was added to stop the kinase reaction. Kinase activity was measured using Lumistar Optima luminometer (BMG LABTECH, Offenburg, Germany). All samples were prepared in triplicates and the assay was repeated at least three times.

### 4.9. Measurement of VEGF Production

VEGF level in DU145 cells with CA and/or CPE and/or SKI and/or NAC was evaluated as previously described [33] by using VEGF ELISA kit (Invitrogen, Carlsbad, CA, USA) according to the manufacturer instructions. Briefly, 50 µL of the culture supernatants was added onto a 96-well microplate, and incubated with 50 µL of dilution buffer and 50 µL of incubation buffer at room temperature for 2 h. The plate was then washed four times with washing buffer and 100 μL of biotin conjugate was placed in each well for 1 h at room temperature. After washing four times with washing buffer, 100 µL of the stabilized chromogen was placed to each well and incubated for 30 min at room temperature in the dark. Finally, 100 µL of stop solution was added to each well and the optical density was measured at 450 nm using microplate reader (Molecular Devices Co., Sunnyvale, CA, USA). All samples were prepared triplicate and the assay was repeated at least three times.

### 4.10. SPHK-1 Gene Silencing

DU145 cells were seed onto 6 well plates and transfected with 100 nM of control siRNA or siRNA SPHK siRNA (Bioneer, Daejeon, Korea) by using INTERFERin transfection reagent (Polyplus, Illkirch, France) for 24 h and treated with or without of CA. After treatment, the cells were confirmed by western blot and Crystal violet staining.

### 4.11. Statistical Analysis

All data were shown as means ± S.D. (standard deviation) of three replications per experiment. In the in vitro experiment, data were analyzed by Student’s *t*-test.

## 5. Conclusions

In summary, we demonstrate that CPE and CA could suppress the activity of SPHK-1 and express HIF-1α, pGSK-3β, and pAKT in hypoxic DU145 cells. SPHK-1 siRNA and SKI augment the inhibitory effect of CA on the accumulation HIF-1α and the expression of p-AKT and pGSK-3β in hypoxic DU145 cells. Furthermore, SKI enhanced anti-angiogenic effect of CA by inhibiting VEGF expression. Furthermore, the anti- proliferative effect of CA involved SPHK-1 pathway. These results suggest that CA inhibits the expression of HIF-1α through suppression of SPHK-1 pathway in hypoxic DU145 cells and thus has an anti-cancer effect.

## Supplementary Materials

Supplementary materials can be found at www.mdpi.com/1422-0067/18/2/325/s1.

## Abbreviations

CA	Chlorogenic acid
CPE	*Crataegus Pinnatifida* Bunge var. typical Schneider ethanol extract
SKI	SPHK inhibitor
SPHK-1	Sphingosine kinase 1
HIF-1α	Hypoxia-inducible factor-1α
PBS	Phosphate-buffered saline
OD	Optical density
