# Supplementary material for: Sphingosine Kinase-1 Involves the Inhibitory Action of HIF-1α by Chlorogenic Acid in Hypoxic DU145 Cells
Source: Int J Mol Sci. 2017 Feb 4;18(2):325. doi: 10.3390/ijms18020325 (PMC5343861; doi:10.3390/ijms18020325)
Supplement: Supplementary file 1 [file ijms-18-00325-s001.pdf]

Supplementary Materials: Sphingosine Kinase-1 Involves the Inhibitory Action of HIF-1α by Chlorogenic Acid in Hypoxic DU145 Cells

Myoung-Sun Lee, Seon-Ok Lee, Kyu-Ri Kim and Hyo-Jeong Lee

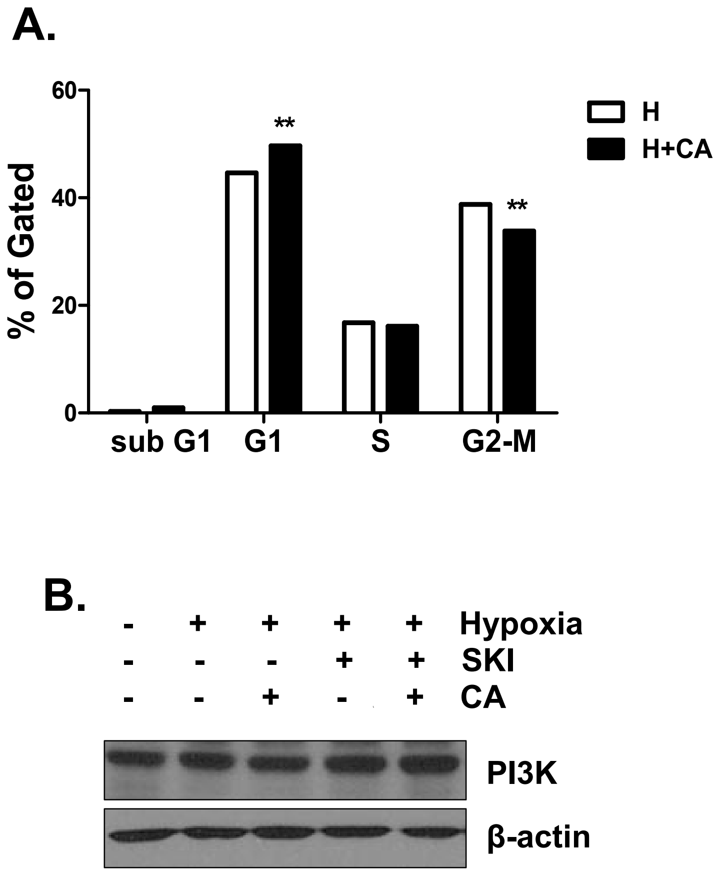

Figure S1. (**A**) CA (50 μM) was treated in hypoxia-induced DU145 cells for 3 days. Cell cycle distribution was analyzed by flow cytometry. Bar graphs represent the percentage of sub-G1, G1, S, G2-M phase cells. Data represent mean ± SD. ** $p < 0.01$ compared with hypoxia control. (H, hypoxia; CA, chlorogenic acid); (**B**) PI3K levels by CA on hypoxia-induced DU145 cells. DU145 cells were treated without or with CA and SKI under hypoxia for 4 h. Levels of PI3K was determined by western blot analysis.
